# Practical Departments

**Published:** 1897-06-05

**Authors:** 


### PRACTICAL DEPARTMENTS.

### TRUSTEES OF COTTAGE HOSPITALS.

The honorary treasurer of the Lyme Regis Cottage Hospital writes for information as to trustees of cottage hospitals and their powers. We have advised him to purchase the third edition of "Cottage Hospitals" (Scientific Press, London, W.C.), which contains full information on this and other points relating to the administration of these institutions.

